# Antigen-specific cytokine profiles for pulmonary *Mycobacterium avium* complex disease stage diagnosis

**DOI:** 10.3389/fimmu.2023.1222428

**Published:** 2023-07-14

**Authors:** Yoshiro Yamashita, Ikkoh Yasuda, Takeshi Tanaka, Toru Ikeda, Mayumi Terada, Masahiro Takaki, Yoshiko Tsuchihashi, Norichika Asoh, Yukiko Ohara, Shymaa Enany, Haruka Kobayashi, Sohkichi Matsumoto, Konosuke Morimoto

**Affiliations:** ^1^ Department of Clinical Medicine, Institute of Tropical Medicine, Nagasaki University, Nagasaki, Nagasaki, Japan; ^2^ Department of Respiratory Medicine, Shunkaikai Inoue Hospital, Nagasaki, Nagasaki, Japan; ^3^ Department of General Internal Medicine and Clinical Infectious Diseases, Fukushima Medical University, Fukushima, Fukushima, Japan; ^4^ Infection Control and Education Center, Nagasaki University Hospital, Nagasaki, Nagasaki, Japan; ^5^ Department of Respiratory Medicine, Nagasaki Rosai Hospital, Sasebo, Nagasaki, Japan; ^6^ Department of Internal Medicine, Koseikai Nijigaoka Hospital, Nagasaki, Nagasaki, Japan; ^7^ Department of Respiratory Medicine, Juzenkai Hospital, Nagasaki, Nagasaki, Japan; ^8^ Department of Bacteriology, Niigata University Graduate School of Medicine, Niigata, Niigata, Japan; ^9^ Department of Microbiology and Immunology, Faculty of Pharmacy, Suez Canal University, Ismailia, Egypt; ^10^ Biomedical Research Department, Armed Force College of Medicine, Cairo, Egypt; ^11^ Department of Respiratory Infectious Disease, Institute of Tropical Medicine, Nagasaki University, Nagasaki, Nagasaki, Japan

**Keywords:** *Mycobacterium avium* complex disease, clinical stage, *Mycobacterium avium*-associated antigens, cell-mediated immunity, CD4+T cells, CD19+B cells, cytokine profile

## Abstract

**Introduction:**

Controlling pulmonary *Mycobacterium avium* complex (MAC) disease is difficult because there is no way to know the clinical stage accurately. There have been few attempts to use cell-mediated immunity for diagnosing the stage. The objective of this study was to characterize cytokine profiles of CD4+T and CD19+B cells that recognize various *Mycobacterium avium*-associated antigens in different clinical stages of MAC.

**Methods:**

A total of 47 MAC patients at different stages based on clinical information (14 before-treatment, 16 on-treatment, and 17 after-treatment) and 17 healthy controls were recruited. Peripheral blood mononuclear cells were cultured with specific antigens (MAV0968, 1160, 1276, and 4925), and the cytokine profiles (IFN-γ, TNF-α, IL-2, IL-10, IL-13, and IL-17) of CD4+/CD3+ and CD19+ cells were analyzed by flow cytometry.

**Results:**

The response of Th1 cytokines such as IFN-γ and TNF-α against various antigens was significantly higher in both the on-treatment and after-treatment groups than in the before-treatment group and control (P < 0.01–0.0001 and P < 0.05–0.0001). An analysis of polyfunctional T cells suggested that the presence of IL-2 is closely related to the stage after the start of treatment (P = 0.0309-P < 0.0001) and is involved in memory function. Non-Th1 cytokines, such as IL-10 and IL-17, showed significantly higher responses in the before-treatment group (P < 0.0001 and P < 0.01–0.0001). These responses were not observed with purified protein derivative (PPD). CD19+B cells showed a response similar to that of CD4+T cells.

**Conclusion:**

There is a characteristic cytokine profile at each clinical stage of MAC.

## 1 Introduction

Pulmonary non-tuberculosis mycobacterial disease (pNTM) is a major form of the non-tuberculosis mycobacterial disease (NTM), and *Mycobacterium avium* complex (MAC) is the most common pathogen (1). NTM is thought to have a comparatively high prevalence in North America and East Asia (2). In Japan, the incidence of pNTM is 14.7 per 100,000 person-years (3), seven times the incidence in the 1990s. In the United States, the incidence in 2015 was 4.73 per 100,000 person-years, and the annual change in incidence was +5.2% (4). The incidence of pNTM in England also rose from 5.6 per 100,000 person-years in 2007 to 7.6 per 100,000 person-years in 2012 (5). Thus, its incidence and associated prevalence are increasing worldwide. Considering that the annual economic burden per patient is estimated to exceed $10,000 (6, 7), the burden of pNTM is getting heavier.

A clinical strategy for controlling pNTM has not yet been established. Because the clinical course of pNTM varies (8, 9), clinicians cannot determine its clinical stage accurately and tend to miss the appropriate timing for starting or finishing antimicrobial therapy. To establish a treatment strategy, the clinical stage of pNTM must be determined accurately.

CD4+T cells, the main casts of cell-mediated immunity, are known to change their properties according to antigen load, specifically, T-helper (Th) 1 (10, 11). Due to their ability to change properties, Th1-CD4+T cells can produce cytokines, such as IFN-γ, TNF-α, and IL-2, either simultaneously or separately. Similar changes have been observed for tuberculosis (TB)-specific Th1-CD4+T cells (12, 13). Antigen load reflects the severity of infection; therefore, the Th1 cytokine profile can vary according to the clinical stage. In addition to Th1, CD4+T cells have subsets such as Th2, Th17, and regulatory T cells (Treg) (14). Because these subsets are thought to cooperate or interfere with each other throughout an infection, they are also expected to show various cytokine production reactions depending on the severity of an infection. Characteristic cytokine profiles for each stage are known in TB (15), but not in MAC disease.

Furthermore, other lymphocytes, such as B cells, assist in CD4+T cell function. Some B cells have memory functions (16). Some B cells produce cytokines (17); however, it is not known whether memory (MAC-specific) B cells produce cytokines.

We postulate that a comprehensive evaluation of CD4+T cell function is needed to determine the clinical stages of MAC. Moreover, information on B cell function complements our understanding of the environment of CD4+T cells in MAC. This study aims to characterize the cytokine profile of CD4+T cells against *Mycobacterium avium* (MA)-associated antigens at different clinical stages of MAC.

## 2 Methods

### 2.1 Study participants

MAC pulmonary disease cases were defined as participants with pulmonary shadows that were consistent with those of pNTM (nodular or cavitary opacities on chest radiograph or a high-resolution computed tomography scan that shows bronchiectasis with multiple small nodules) and with more than one positive finding of culture or PCR analyses from sputum or bronchoalveolar lavage fluid or more than one positive finding of blood IgA antibody against glycopeptidolipid-core antigen (18, 19). Treatment was performed according to the guidelines of the Japanese Society for Tuberculosis and Non-tuberculous Mycobacteriosis, and there were no retreatment cases. Healthy controls were defined as participants with no history of MAC and no suspicions of pNTM.

Participants were recruited from Nagasaki University Hospital, Nagasaki Rosai Hospital, Shunkaikai Inoue Hospital, Kosei-kai Nijigaoka Hospital, and Juzenkai Hospital. All blood samples included in this study were drawn after obtaining informed consent from participants and ethical approval from the Institute of Tropical Medicine and Nagasaki University Joint Ethics Committee (number: 191003222).

The participants were divided into three study groups (before-treatment, on-treatment, and after-treatment) based on the clinical information. Before-treatment cases were defined as those who had never been treated with drugs (before initiation of drug treatment). On-treatment cases were defined as those who were undergoing drug treatment when the blood samples were collected, and after-treatment cases were defined as those who had completed drug treatment. Finally, we compared outcomes between the three study groups and the control group.

### 2.2 Reagents

MAV0986, MAV1160, MAV1276, and MAV4925 are recombinant protein products of MA. MAV1160 is a diaminopimelic acid decarboxylase. MAV1276 is an acetyl-CoA acetyltransferase. MAV4925 is a glucose-methanol-choline oxidoreductase. However, the function of MAV0986 remains unclear. The synthesis of these proteins was carried out as described in the online supplement.

The fluorescently labeled monoclonal antibodies used in this study are also shown in the online supplement.

### 2.3 *In vitro* peripheral blood mononuclear cell culture and antigen stimulation

Peripheral blood mononuclear cell isolation and culture were performed as described previously (20). Cells were isolated within 8 h of obtaining heparinized blood samples. Each MA-associated antigen concentration was 2.1 µg/ml. Staphylococcus endotoxin b (15 μg/ml) and Purified Protein Derivative (25 μg/ml) were used as positive controls. Cultures without antigens (as negative control) were also included. All antigen stimulations were performed in the presence of CD28/CD49d co-stimulator (0.5 µg/ml) and Golgi blocker: brefeldin-A (1 µg/ml) and monensin (0.5 µM). Cell incubation was overnight at 37 °C in a 5% CO_2_ incubator.

### 2.4 Cell staining and flow cytometry analyses

Cell surface staining, permeabilization, and intracellular cytokine staining were performed as described previously (20). For surface staining, 30% goat serum containing anti-CD3, anti-CD4, anti-CD19, FcR blocking reagent, and LIVE/DEAD reagent was added. For intracellular cytokine staining, an anti-cytokine monoclonal antibody cocktail and FcR-blocking reagent were added. Cells were acquired using Gallios (Beckman Coulter). Flow cytometry data were analyzed using FlowJo software, version 10 (TreeStar, San Carlos, CA, USA). The gating strategy for flow cytometric analysis is shown in **Supplementary Figure 2**.

### 2.5 Statistical analysis

Group medians and distributions were analyzed using the Kruskal-Wallis test with post-hoc Dunn’s comparison test. The medians and distributions of the two groups were analyzed using the Mann-Whitney U test. The group ratios were analyzed using the chi-square test. Correlations were analyzed using the Spearman correlation test. Analyses were performed using GraphPad Prism software version 7 (GraphPad Software, San Diego, CA, USA). The threshold for significance was set at P < 0.05.

## 3 Results

### 3.1 Study participants

A total of 47 MAC cases and 18 healthy controls were recruited for this study. One control was excluded due to impaired blood sample quality. Subsequently, 14 before-treatment, 16 on-treatment (median duration of treatment —13 months), 17 after-treatment cases (median duration after completion of treatment — 62 months), and 17 healthy controls were evaluated.

None of the participants were diagnosed with HIV or other immunodeficiencies. The characteristics of the participants are presented in **Table 1**. There were no significant differences in median age and BMI between the groups. In addition, there were no significant differences in the percentages of men and selected clinical histories (diabetes, malignancy, chronic respiratory disease, immunosuppressant use, and smoking) between the groups.

**Table 1:** Characteristics of the participants.

Characteristic	Before-treatment (n=14)	On-treatment (n=16)	After-treatment (n=17)	Control (n=17)	Difference
Age-yr					
Median	70.0	69.5	72.0	73.0	P=0.7310 †
Range	47.0-89.0	53.0-84.0	51.0-84.0	49.0-86.0	
**Male sex-no.(%)**	3 (17.6)	5 (31.3)	3 (16.7)	4 (22.2)	P=0.6062 ††
BMI					
Median	20.3	19.7	20.2	21	P=0.2548 †
Range	16.2-27.8	13.4-26.0	14.7-25.4	18.9-31.0	
Clinical History-no. (%)					
Diabetes	1 (5.6)	4 (25.0)	0 (0)	2 (11.8)	P=0.1018 ††
Malignancy	1 (5.6)	1 (6.3)	4 (22.2)	2 (11.8)	P=0.4023 ††
Chronic respiratory dis	7 (41.2)	3 (18.8)	6 (33.3)	7 (41.2)	P=0.4849 ††
Immunosuppressant use	4 (22.2)	2 (12.5)	4 (22.2)	5 (29.4)	P=0.7040 ††
Smoking	2 (11.1)	6 (37.5	3 (16.7)	5 (29.4)	P=0.2791 ††

The differences between each set of samples were assessed using † the Kruskal-Wallis test and *post hoc* Dunn’s comparison test and †† Chi-square test. P values less than 0.05 were interpreted as statistically significant.

### 3.2 Cytokine response of CD4+T cells to a range of MA-associated antigens

**Figure 1:**
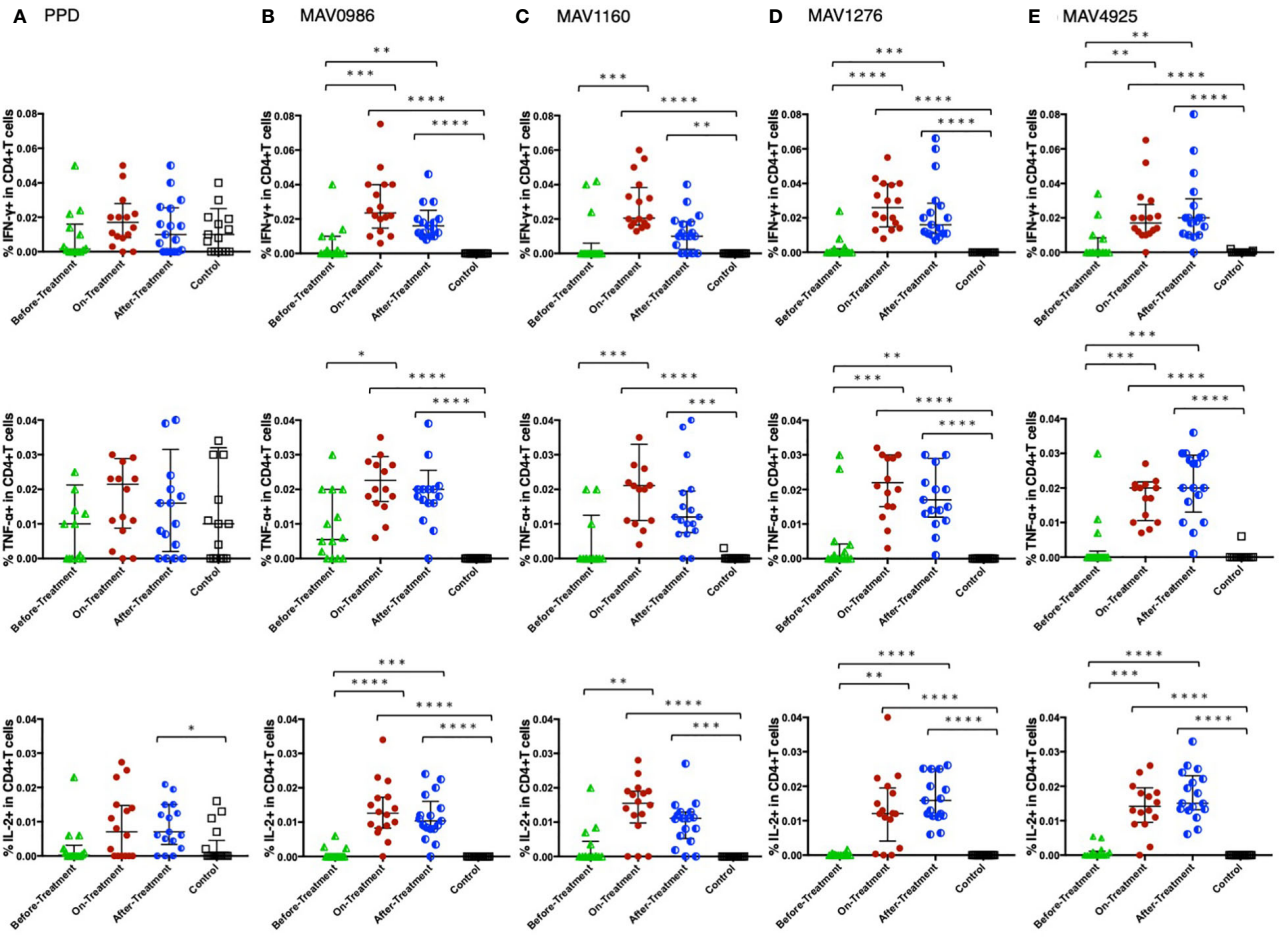
Th1 cytokine (IFN-γ, TNF-α, and IL-2) response of CD4+T cells to MA-associated antigens. Th1 cytokine responses of CD4+T cells in MAC cases grouped according to disease stage as before-treatment MAC cases (n=14), on-treatment MAC cases (n=16), and after-treatment MAC cases (n=17). The responses of the control group (n=17) are also shown. The differences in each set of samples were assessed using the Kruskal-Wallis test and *post hoc*Dunn’s comparison test (*P<0.05, **P<0.01, ***P<0.001, and ****P<0.0001). The long horizontal line represents the median and the vertical line represents the interquartile range. **(A)** Cytokine responses to PPD. Three data points are outside the limits in IFN-γ, nine data points in TNF-α, and one data point in IL-2. **(B)** Cytokine responses to the MA-associated antigen: MAV0986. One data point in IFN-γ and four data points in TNF-α are outside the limits. **(C)** Cytokine responses to the MA-associated antigen: MAV1160. Four data points are outside the limit in TNF-α. **(D)** Cytokine responses to the MA-associated antigen: MAV1276. Four data points in TNF-α and one data point in IL-2 are outside the limits. **(E)** Cytokine responses to the MA-associated antigen: MAV4925. Two data points in TNF-α and one data point in IL-2 are outside the limits.

**Figure 1** shows the Th1 cytokine (IFN-γ, TNF-α, and IL-2) responses of CD4+T cells to a range of MA-associated antigens in before-treatment, on-treatment, and after-treatment groups compared to that of the control group. When stimulated with MA-associated antigen instead of purified protein derivative (PPD), the groups exposed to the treatment (on-treatment and after-treatment) showed significantly higher Th1 cytokine responses than the controls. These groups also showed higher Th1 cytokine responses than the before-treatment group. Although no significant differences were observed, IL-2 responses to MAV1276 and MAV4925 tended to increase in the after-treatment group.

**Figure 2:**
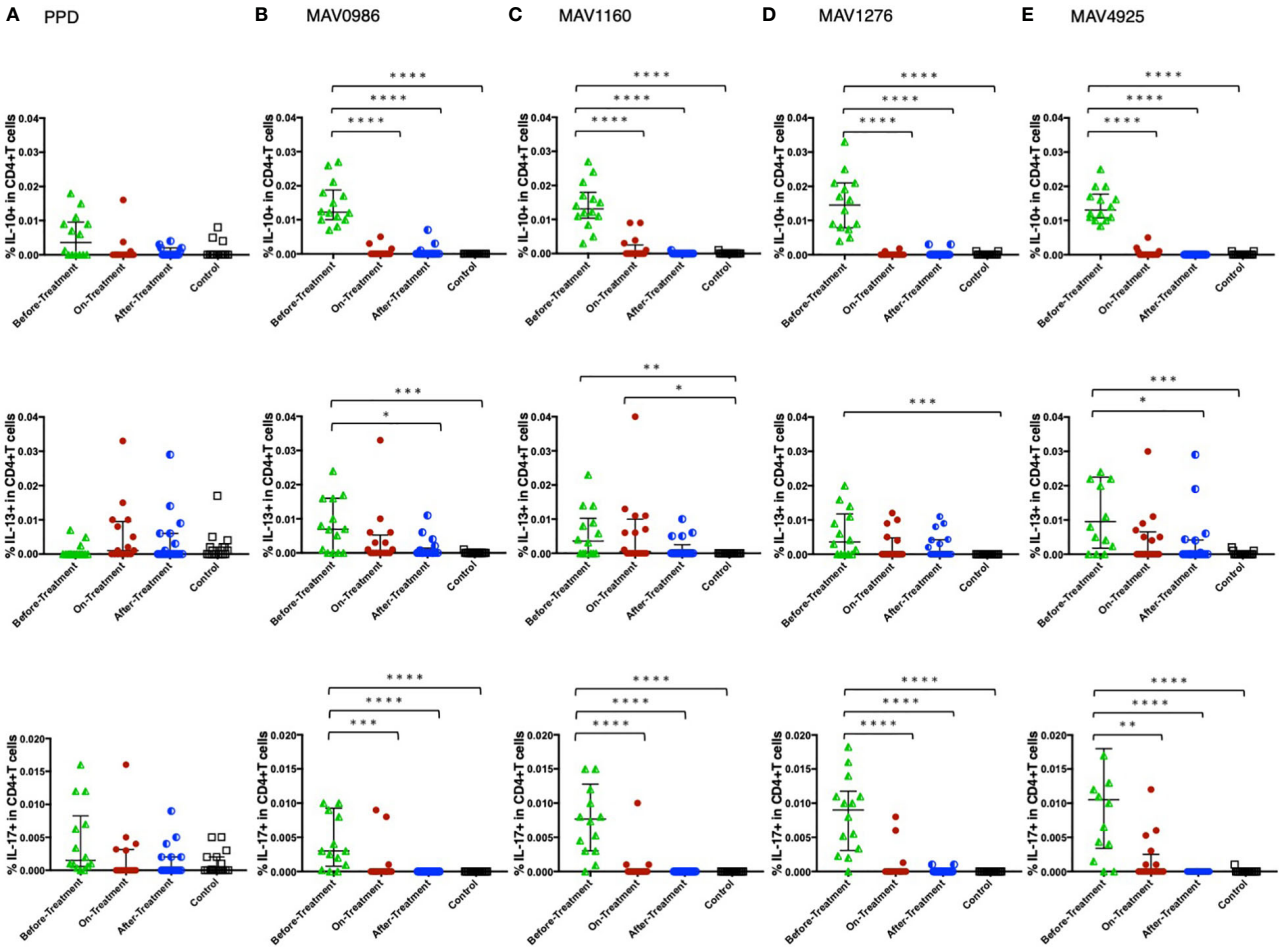
Non-Th1 cytokine (IL-10, IL-13, and IL-17) responses of CD4+T cells to MA-associated antigens. Non-Th1 cytokine responses of CD4+T cells in MAC cases, grouped according to disease stage as before-treatment MAC cases (n=14), on-treatment MAC cases (n=16), and after-treatment MAC cases (n=17). The responses of the control group (n=17) are also shown. The differences in each set of samples were assessed using the Kruskal-Wallis test and *post hoc*Dunn’s comparison test (*P<0.05, **P<0.01, ***P<0.001, and ****P<0.0001). The long horizontal line represents the median and the vertical line represents the interquartile range. **(A)** Cytokine responses to PPD. One data point is outside the limits of IL-10. **(B)** Cytokine responses to the MA-associated antigen: MAV0986. One data point is outside the limits in IL-17. **(C)** Cytokine responses to the MA-associated antigen: MAV1160. One data point is outside the limits in IL-17. **(D)** Cytokine responses to the MA-associated antigen: MAV1276. **(E)** Cytokine responses to the MA-associated antigen: MAV4925. Two data points in IL-13 and three data points in IL-17 are outside the limits.

**Figure 2** shows the non-Th1 cytokine (IL-10, IL-13, and IL-17) responses of CD4+T cells to a range of MA-associated antigens in the before-treatment, on-treatment, and after-treatment groups compared to that of the control group. IL-10 and IL-17 responses were significantly higher in the before-treatment group than in the other three groups although high IL-17 responses were observed in some participants in the on-treatment group. The tendency of the IL-17 response was similar to that of the IL-10 response. The tendency of the IL-13 response was quite different from that of the other two cytokine responses. IL-13 responses were significantly higher in the before-treatment group.

### 3.3 Interaction between cytokines in response to MA-associated antigens

**Figure 3:**
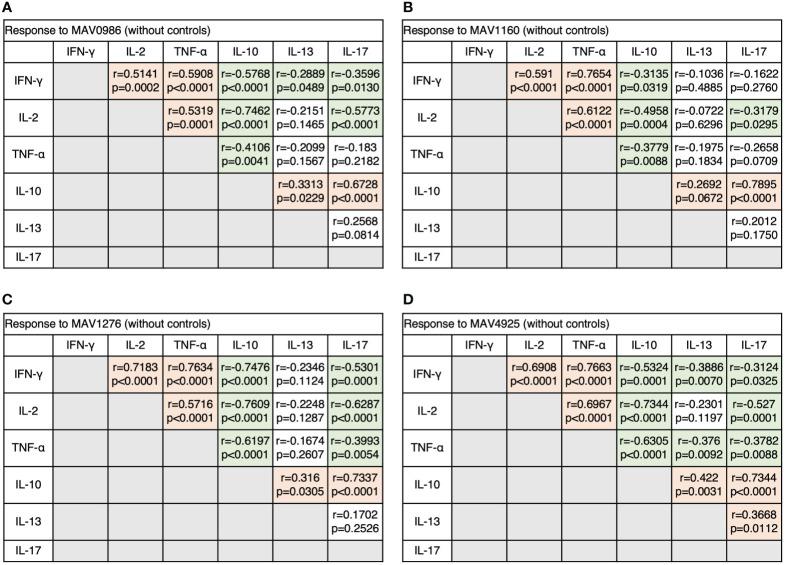
The correlations between cytokines of CD4+T cells in response to MA-associated antigens among MAC cases. The correlations between each cytokine were assessed using the Spearman correlation test. **(A)** Against MAV0986. **(B)** Against MAV1160. **(C)** Against MAV1276. **(D)** Against MAV4925.

Next, we analyzed the correlation between diverse cytokine responses upon stimulation with different MA-associated antigens among MAC cases (**Figures 3A–D**). Th1 cytokines positively correlated with other Th1 cytokines and non-Th1 cytokines positively correlated with other non-Th1 cytokines: IFN-γ and TNF-α showed relatively strong correlations (r = 0.5908–0.7663), and IL-10 and IL-17 generally showed strong correlations (r = 0.6728–0.7895). Furthermore, inverse correlations were observed between Th1 and non-Th1 cytokines; in particular, relatively strong inverse correlations were observed between IL-10 and Th1 cytokines (r = -0.3135 – -0.7609). However, IL-13 was often noted to weakly correlate with Th1 cytokines (r = -0.2889 – -0.3886). The results showed that non-Th1 cytokines, especially IL-10, tended to respond separately from Th1 cytokines. Therefore, clinical stages of MAC in which Th1 or non-Th1 cytokine profile is predominant were suggested.

### 3.4 Polyfunctionality of CD4+T cells

**Figure 4:**
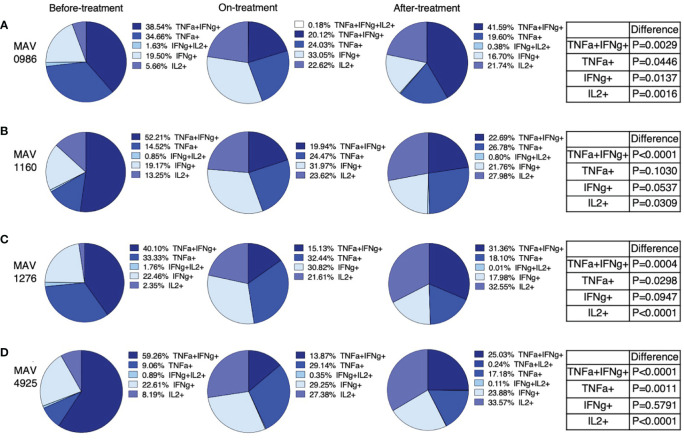
Polyfunctionality analysis of Th1 cytokine (IFN-γ, TNF-α, and IL-2) producing CD4+T cells in MAC cases, grouped according to disease stage as before-treatment cases, on-treatment cases, and after-treatment cases. Triple cytokine (IFN-γ, TNF-α, and IL-2) positive cells, double cytokine (two kinds of IFN-γ, TNF-α, and IL-2) positive cells, and single-cytokine positive cells were detected by using FlowJo^®^ software. Then percentages of these cells in whole cytokine (IFN-γ, TNF-α, and IL-2) positive cells were calculated and shown in the pie chart. Differences in the percentage of cytokine-positive cells in each set of samples were assessed using the Chi-square test. The threshold for significance was P < 0.05. **(A)** MAV0986. **(B)** MAV1160. **(C)** MAV1276. **(D)** MAV4925.

We extracted polyfunctional cells using flow cytometry and calculated the mean of these cells. Furthermore, the total of the means was taken as 100% and the percentage of each polyfunctional cell was investigated. **Figure 4** shows the percentages of polyfunctional Th1 cytokine-producing CD4+T cells. Polyfunctional cells were mainly composed of four types of cells: TNF-α and IFN-γ double-positive (TNF-α+IFN-γ+), TNF-α single-positive (TNF-α+), IFN-γ single-positive (IFN-γ+), and IL-2 single-positive (IL-2+). Considering the combination of TNF-α+IFN-γ+ and TNF-α+ as a whole, TNF-α-positive cells and TNF-α+IFN-γ+ tended to account for a large proportion of the before-treatment group. IFN-γ+ tended to account for large proportions of the on-treatment group, and IL-2+ tended to account for a large proportion of groups after treatment initiation.

**Figure 5:**
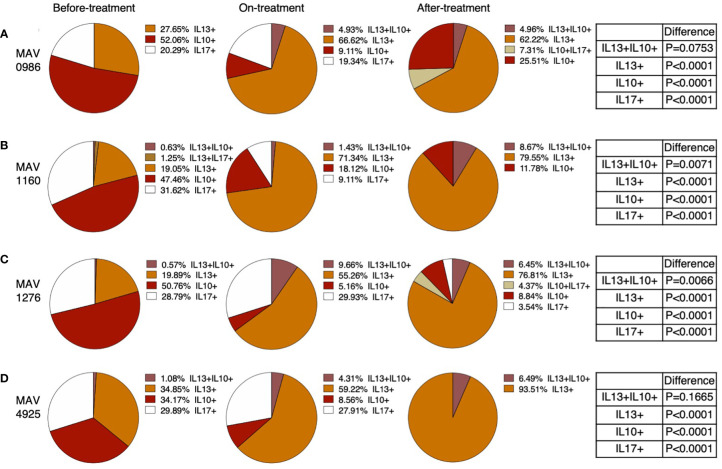
Polyfunctionality analysis of non-Th1 cytokine (IL-10, IL-13, and IL-17) producing CD4+T cells in MAC cases, grouped according to disease stage as before-treatment cases, on-treatment cases, and after-treatment cases. Triple cytokine (IL-10, IL-13, and IL-17) positive cells, double cytokine (two kinds of IL-10, IL-13, and IL-17) positive cells, and single cytokine-positive cells were detected by using FlowJo^®^ software. Then percentages of these cells in whole cytokine (IL-10, IL-13, and IL-17) positive cells were calculated and shown in a pie chart. Differences in the percentage of cytokine-positive cells in each set of samples were assessed using the Chi-square test. The threshold for significance was P < 0.05. **(A)** MAV0986. **(B)** MAV1160. **(C)** MAV1276. **(D)** MAV4925.

**Figure 5** shows the percentage of polyfunctional cells in non-Th1 cytokine-producing CD4+T cells. Although small proportions of IL-13 and IL-10 double positive (IL-13+IL-10+) were observed, most of the non-Th1 cytokine-producing CD4+T cells were composed of single cytokine-positive cells. IL-10 single-positive (IL-10+) cells were predominantly observed in the before-treatment group. IL-10+ proportion was reduced after the start of treatment; however, IL-10+ was maintained at a certain proportion in the after-treatment group. The proportion of IL-17 single-positive (IL-17+) cells also tended to resemble the proportion of IL-10+ cells where almost no IL-17+ cells were found in the after-treatment group. IL-13 single-positive (IL-13+) cells were found at a certain proportion before treatment, but the proportion of IL-13+ cells increased after the start of treatment. It was also found that the composition of cytokine-producing cells differed depending on the clinical stage.

### 3.5 Cytokine-producing CD19+B cells against MA-associated antigens

**Figure 6:**
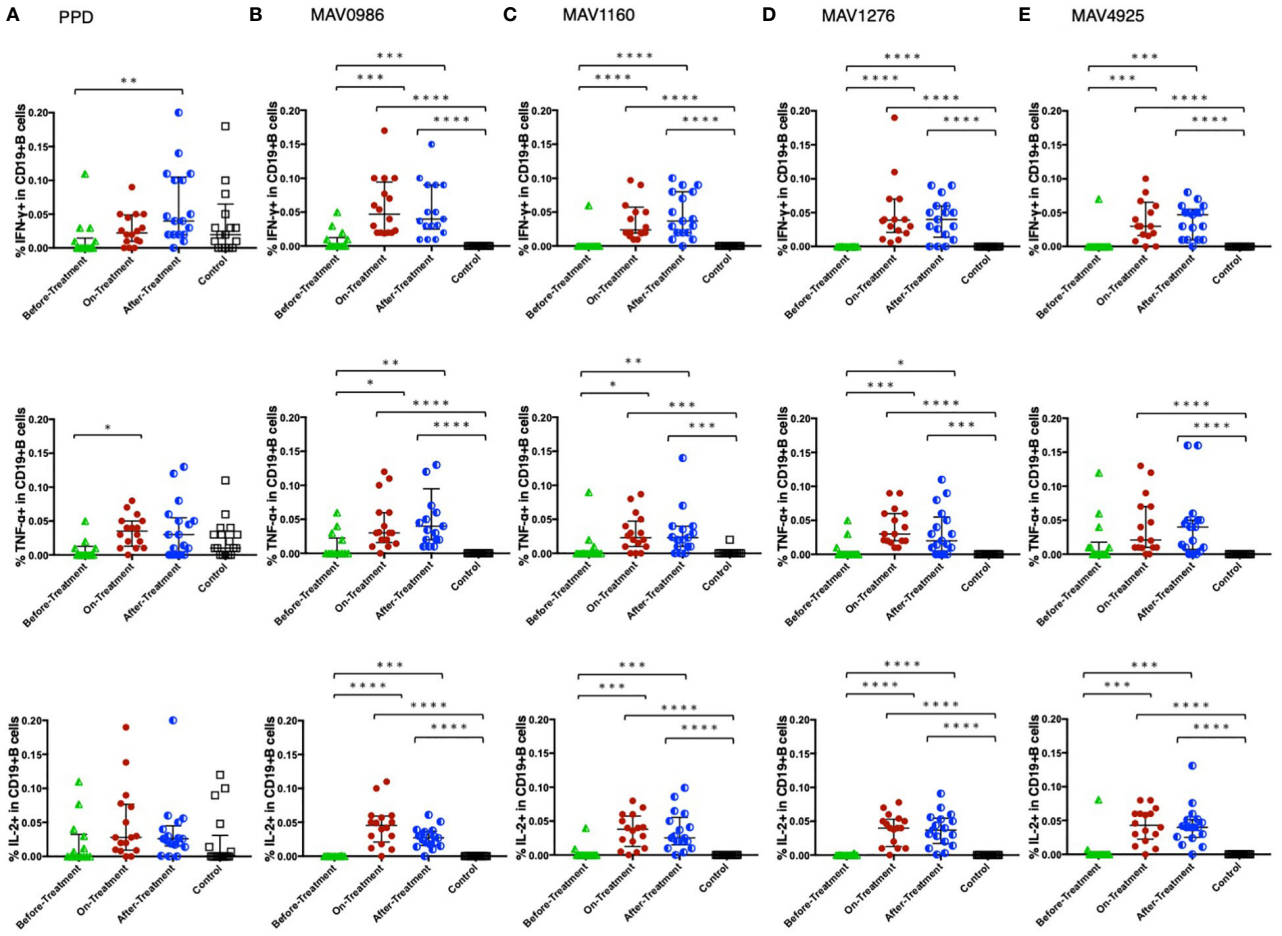
Th1 cytokine (IFN-γ, TNF-α, and IL-2) responses of CD19+B cells to MA-associated antigens. Th1 cytokine responses of CD19+B cells in MAC cases, grouped according to disease stage as before-treatment MAC cases (n=14), on-treatment MAC cases (n=16), and after-treatment MAC cases (n=17). The responses of the control group (n=17) are also shown. The differences in each set of samples were assessed using the Kruskal-Wallis test and *post hoc*Dunn’s comparison test (*P<0.05, **P<0.01, ***P<0.001, and ****P<0.0001). The long horizontal line represents the median and the vertical line represents the interquartile range. **(A)** Cytokine responses to PPD. One data point in IFN-γ and one data point in TNF-α are outside the limits. **(B)** Cytokine responses to the MA-associated antigen: MAV0986. One data point in IFN-γ and two data points in TNF-α are outside the limits. **(C)** Cytokine responses to the MA-associated antigen: MAV1160. One data point in IFN-γ and one data point in TNF-α are outside the limits. **(D)** Cytokine responses to the MA-associated antigen: MAV1276. One data point is outside the limits in IFN-γ. **(E)** Cytokine responses to the MA-associated antigen: MAV4925. One data point is outside the limits in IFN-γ.

**Figure 7:**
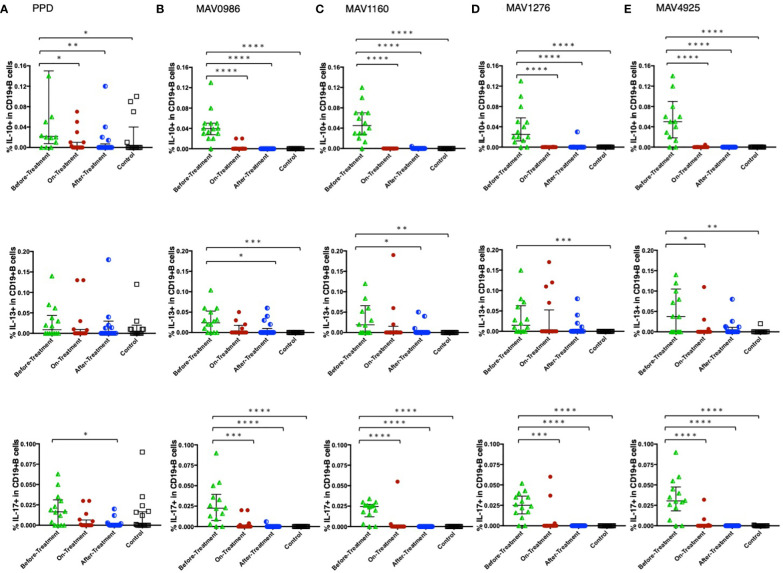
Non-Th1 cytokine (IL-10, IL-13, and IL-17) responses of CD19+B cells to MA-associated antigens. Non-Th1 cytokine responses of CD19+B cells in MAC cases, grouped according to disease stage as before-treatment MAC cases (n=14), on-treatment MAC cases (n=16), and after-treatment MAC cases (n=17). Responses of the control group (n=17) are also shown. The differences in each set of samples were assessed using the Kruskal-Wallis test and *post hoc*Dunn’s comparison test (*P<0.05, **P<0.01, ***P<0.001, and ****P<0.0001). The long horizontal line represents the median and the vertical line represents the interquartile range. **(A)** Cytokine responses to PPD. Four data points in IL-10 and four data points in IL-13 are outside the limits. **(B)** Cytokine responses to the MA-associated antigen: MAV0986. One data point is outside the limits of IL-13. **(C)** Cytokine responses to the MA-associated antigen: MAV1160. Two data points are outside the limits in IL-13. **(D)** Cytokine responses to the MA-associated antigen: MAV1276. **(E)** Cytokine responses to the MA-associated antigen: MAV4925. One data point in IL-10 and one data point in IL-13 are outside the limits.

**Figure 8:**
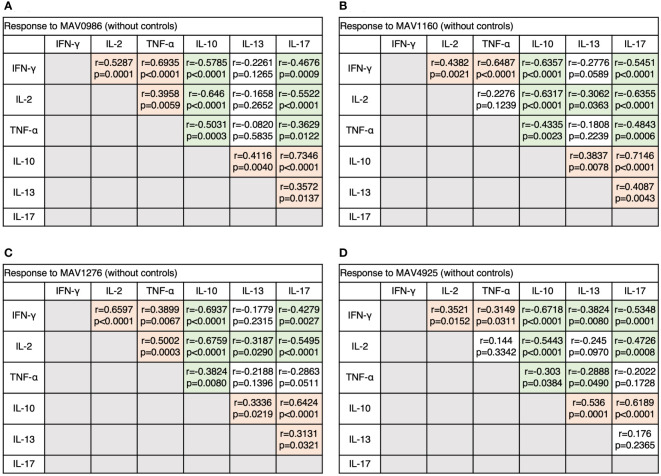
The correlations between cytokines of CD19+B cells in response to MA-associated antigens among MAC cases. The correlations between each cytokine were assessed using the Spearman correlation test. **(A)** Against MAV0986. **(B)** Against MAV1160. **(C)** Against MAV1276. **(D)** Against MAV4925.

**Figure 6** shows the Th1 cytokine responses of CD19+B cells to a range of MA-associated antigens in the before-treatment, on-treatment, after-treatment, and healthy control groups. CD19+B cells also produce cytokines against MA-associated antigens. Higher Th1 cytokine responses were observed in groups after treatment initiation. **Figure 7** shows the non-Th1 cytokine response of CD19+B cells to a range of MA-associated antigens. Higher non-Th1 response rates were observed in the before-treatment group. Notably, the cytokine production pattern exhibited by CD19+B cells at each clinical stage was similar to that exhibited by the CD4+T cells. Correlation analysis also showed results similar to those for CD4+T cells (**Figure 8**). Through the analysis of CD19+B cells, we found that there are MAC-specific CD19+B cells that produce cytokines, like CD4+T cells, and the cytokine profile of MAC-specific CD19+B cells is also different at each clinical stage.

### 3.6 Detailed changes at each clinical stage

**Figure 9:**
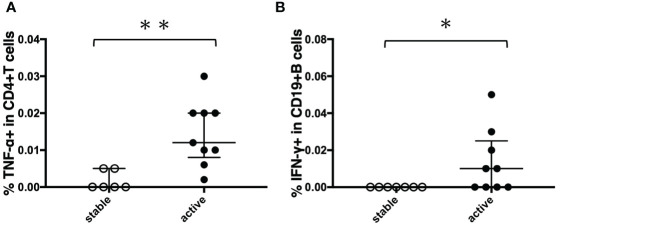
Comparison of cytokine responses between stable cases and unstable cases in before-treatment MAC cases. Before-treatment MAC cases were divided into stable cases and active cases according to clinical information. The difference between the two sets of samples was assessed using the Mann-Whitney test (*P<0.05 and **P<0.01). The long horizontal line represents the median and the vertical line represents the interquartile range. **(A)** TNF-α responses of CD4+T cells against MA-associated antigen: MAV0986. **(B)** IFN-γ responses of CD19+B cells against MA-associated antigen: MAV0986.

**Figure 10:**
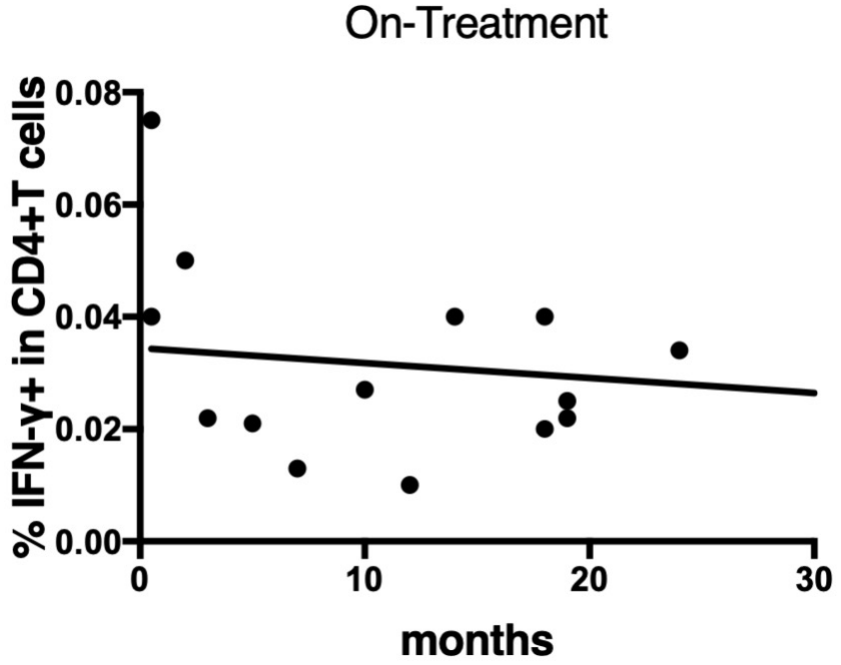
Linear regression between IFN-γ responses of CD4+T cells against MA-associated antigen, MAV0986, and elapsed time from the start of treatment in on-treatment MAC cases. The slope is significantly non-zero (y = -0.0002637x + 0.0344; P = 0.0464).

**Figure 11:**
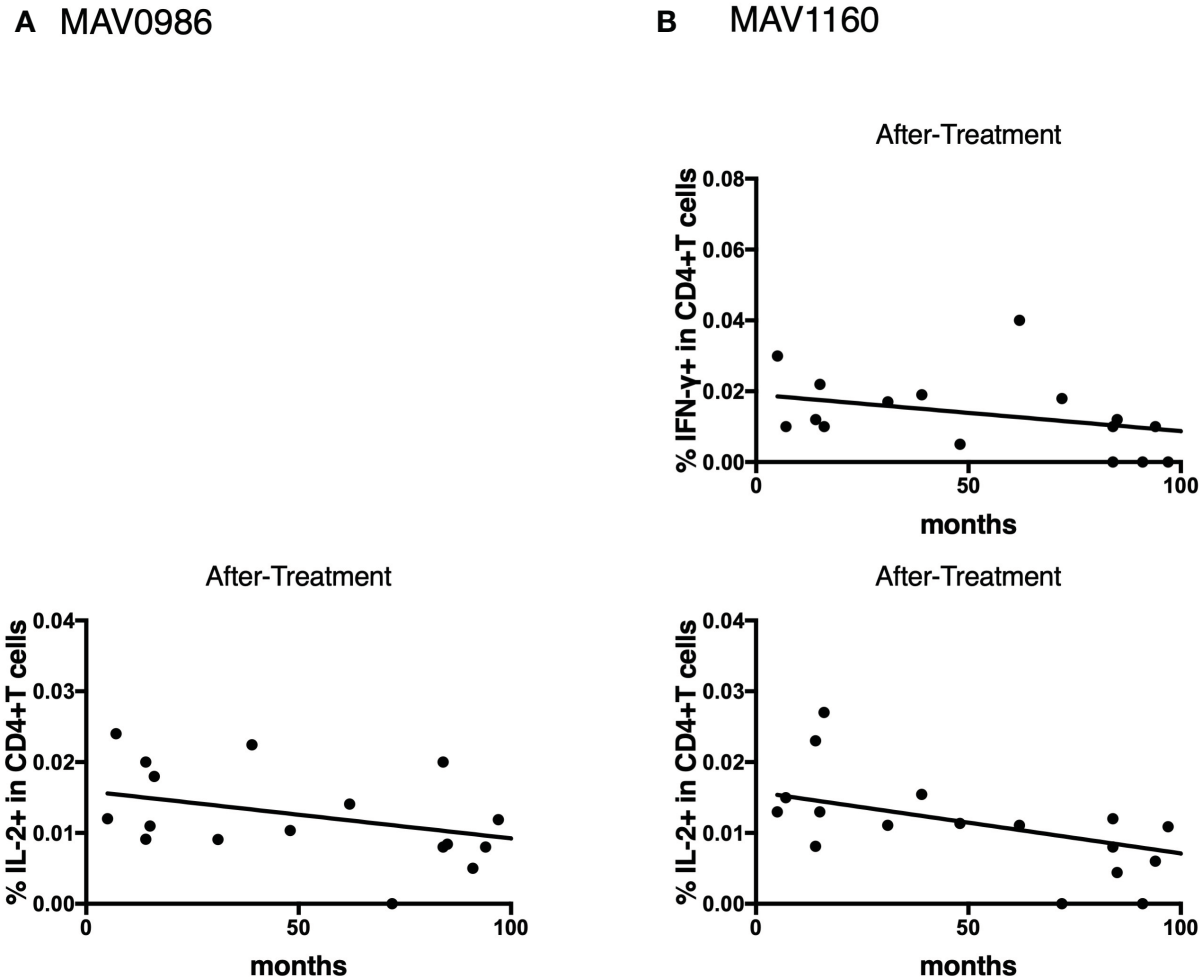
Linear regression between cytokine responses of CD4+T cells against MA-associated antigens and elapsed time from the end of treatment in after-treatment MAC cases. **(A)** against MAV0986. **(B)** against MAV1160. These slopes are significantly non-zero (IL-2 against MAV0986: y = -0.00006137x + 0.01529; P = 0.0477, IFN-γ against MAV1160: y = -0.0001034x + 0.01909; P = 0.0484, and IL-2 against MAV1160: y = -0.0000774x + 0.01472; P = 0.0093).

To determine the state of the disease in more detail, we analyzed cytokine differences at each clinical stage. **Figure 9** shows the cytokine production differences in the before-treatment group. We divided the before-treatment group into stable and active groups according to clinical information, i.e., whether there have been any changes in imaging findings within the last six months from the sample collection date, and we then compared these two groups. Th1 cytokines such as TNF-α in CD4+T cells stimulated with MAV0986 and IFN-γ in CD19+B cells stimulated with MAV0986 showed significant differences (P = 0.0016 and P = 0.0337, respectively). We expected that under stimulation with some MA-associated antigens, Th1 cytokines would be associated with their production and elapsed time. For the on-treatment group, we attempted a linear regression between the cytokine production response and the elapsed time from the start of treatment. **Figure 10** shows that IFN-γ in CD4+T cells stimulated with MAV0986 had a significantly non-zero slope (y = -0.0002637x + 0.0344; P = 0.0464). **Figure 11** shows the results of the linear regression analysis in the after-treatment group. We also attempted a linear regression between the cytokine production response and the elapsed time from the end of treatment. Similar to on-treatment, IFN-γ levels in CD4+T cells stimulated with MAV1160 tended to decrease over time after treatment (y = -0.0001034x + 0.01909; P = 0.0484). However, changes in IL-2 were noticeable after treatment (IL-2 in CD4+T cells stimulated with MAV0986: y = -0.00006137x + 0.01529; P = 0.0477, and IL-2 in CD4+T cells stimulated with MAV1160: y = -0.0000774x + 0.01472; P = 0.0093).

## 4 Discussion

In the current study, we showed that the cytokine profile of CD4+T cells against a wide range of pNTM-associated antigens is different at each clinical stage of MAC. Th1 cytokine levels were elevated after the start of treatment; whereas, non-Th1 cytokine levels were elevated before treatment. Additionally, CD19+B cells showed cytokine profiles similar to those of CD4+T cells at each stage.

Among Th1 cytokines, IFN-γ and TNF-α showed a tendency to show high responses in the on-treatment group and the active group (a subgroup of the before-treatment group); these groups were expected to have high mycobacterial activity. A case of disseminated MAC positive for anti-IFN-γ autoantibodies has been reported (21). In addition, IFN-γ gene expression has been reported to be reduced in pNTM patients compared to that in uninfected patients (22). Furthermore, anti-TNF-α therapy was reported to be associated with an increased risk of NTM (23). These studies suggest that IFN-γ and TNF-α play important roles in protection against MAC, and the results of the current study are consistent with this. IFN-γ signals from CD4+T cells led to the activation of CD8+T cells and reduced the burden of *Mycobacterium tuberculosis* (MTB) in macrophages (24). TNF-α signaling was required for nitric oxide production in macrophages, and nitric oxide was useful for digesting phagocytic mycobacteria (25). Thus, their role is to activate immune cells such as macrophages and cytotoxic T cells. Paradoxically, high responses to these cytokines would mean that the amount of MAC is high. Because of the large amounts of MAC and the high exposure of T cells to all the antigens that make up MAC, the tendency of these cytokine responses was similar for all four antigens.

IL-2 response increased in the groups after the start of treatment. IL-2 production by undifferentiated T cells with antigens suggests a very early stage of infection (26). Upon further stimulation with IL-2, these T cells differentiate into CCR7+ effector memory T cells (27). T cells can be stimulated by IL-2 in both an autocrine and paracrine manner (28). Thus, T cells may become one of the major sources of IL-2 during late infection, as shown by the aforementioned results. MAV1276 and MAV 4925 are considered proteins necessary for survival in the host. A small number of mycobacteria are expected to remain hidden in the host even after treatment. The reason why the response of IL-2 against these antigens increased in the after-treatment group is thought to be that memory T cells reacted to these antigens, which may work quietly to survive. In addition, the results of this study showed that the IL-2 response decreased with the passage of time from the end of treatment. A decrease in IL-2 signaling results in a decrease in the number of memory T cells (29–31).

Polyfunctional T cells are considered excellent in terms of both the quality and quantity of cytokine secretion (32). When the requirement for IFN-γ and TNF-α increases, these polyfunctional T cells are expected to play an active role. However, in this study, the proportion of polyfunctional T cells was reduced in the on-treatment group. This result suggests that polyfunctional T cells play a role as a reserve rather than acting directly as a defense mechanism. It is expected that single-positive cells for IFN-γ or TNF-α, which increase the proportion of polyfunctional T cells, are terminally differentiated cells (33) and directly involved in protection.

Few studies have described the association between IL-13 and MAC and NTM infections (34, 35). IL-13 has been suggested to protect against infection by promoting cell turnover and excreting infected epithelial cells (36). Some mycobacteria, such as *Mycobacterium leprae* are thought to invade alveolar epithelial cells through adhesion molecules (37). Because MAC is also expected to invade epithelial cells through adhesion molecules (38, 39), IL-13 production continues as long as the invasion continues. This was shown in the aforementioned results, in the before-treatment group to the on-treatment group.

The production of IL-10 in MAC infection occurred before treatment, like in TB cases (15). IL-10 has been shown to suppress host immunity even in mycobacteria infections (40). It has been suggested that proteins that make up the cell wall of mycobacteria may promote the production of IL-10 from antigen-presenting cells and T lymphocytes (41). In MTB*-*infected cells, the MTB gene stimulates the production of type I IFN, such as IFN-β, resulting in increased IL-10 production from neighboring cells (42). Prostaglandin E2 is a bioactive substance included within the arachidonic acid cascade and is known to stimulate IL-10 (43) when mycobacteria invade the epithelial cells. It is possible that mycobacteria create a favorable environment for their own survival through the action of IL-10 immediately after invading the host body.

IL-17 production in MAC infection was also observed in before-treatment cases, as in TB (15). The growth of mycobacteria in the host purely increases TCR stimulation, which leads to increased T-bet, a master regulator of Th1 expression (44, 45). Low T-bet expression appears to create an environment in which Th17 cells are dominant (46). The environment in which Th17 is dominant often seems to work disadvantageously in defense against intracellular pathogens (47). In contrast, IL-17-producing CD4+T cells, regarded as Th17 cells, are reported to recruit IFN-γ-producing CD4+T cells, regarded as Th1 (48). Therefore, the role of IL-17 in mycobacterial infections may be to prime Th1 cell establishment rather than protection.

The responses of non-Th1 cytokines did not differ greatly between the antigens in the aforementioned results. Thus, the four peptides examined in this study may have similar functions. In TB, the presence of antigens that strongly stimulate non-Th1 cytokine responses has been observed in before-treatment cases (15). MTB may contain specific peptides that can be adapted to humans. If such peptides exist in MAC and can be added, a cytokine profile different from the aforementioned results may be observed.

The dynamics of antigen-specific cytokine-producing B cells resemble those of T cells, suggesting a relationship between the two. The long-known function of B cells—antigen presentation—is the function that connects B cells and T cells (49). Considering this function, B cells may be activated during an earlier stage than T cells. Antigen-specific B cell involvement has also been shown in mycobacterial infections, and these B cells are suggested to produce various cytokines by signals from T cells (50). If so, B cells may be activated later than T cells. However, because memory B cells may be activated via pattern recognition molecules (49), the exchange of signals between B cells and T cells is not one-way but may be mutual.

The following cell-mediated immune dynamics can be inferred based on the results of this study. Shortly after MAC invasion, the non-Th1 subsets of CD4+T cells will be in harmony. At local areas such as the airway epithelium, differentiation into Th2 or Th17 will start. MAC may induce IL-10-producing T cells for survival. When MAC proliferates and the momentum of the disease becomes active, the Th1 subset becomes dominant for protection against MAC. Th1 cells then establish an immune memory for MAC by producing IL-2. If a decrease in IL-2 levels is observed, the risk of relapse might increase. Paradoxically, by monitoring the response of immune cells or alternatively, by analyzing the cytokine profile, we would be able to identify the clinical stage of MAC disease. This will also lead to the early diagnosis of MAC and the evaluation of therapeutic effects.

This study had some limitations. First, we collected all samples within a defined period, but the number of participants was still not large enough to set up a group like in a previous study (15). It may have been possible to analyze the transition of the cytokine profile in more detail if we could set up a group before and immediately after the start of treatment. Second, this study was a cross-sectional study. The ability to follow the cytokine profile longitudinally would have produced more robust results. Finally, fresh samples were used because of the past verification (20). To keep the samples fresh, the range of transfer was limited; as a result, it is possible that the sample was taken only from a population with similar characteristics. In addition, because the number of samples that could be processed at once was limited, the different conditions depending on the day, such as temperature, might have affected the sample processing.

In conclusion, it has become clear that there are different cytokine profiles specific to each clinical stage of MAC, demonstrated using MAC-specific antigens. The results of this study can be applied to the development of diagnostic tools which will greatly benefit the management of MAC.

## Data availability statement

The datasets generated for this study are available on reasonable request to the corresponding author.

## Ethics statement

The studies involving human participants were reviewed and approved by the Institute of Tropical Medicine and Nagasaki University Joint Ethics Committee (number: 191003222). The patients/participants provided their written informed consent to participate in this study.

## Author contributions

YY performed laboratory investigations, designed the study, analyzed the data, and prepared the manuscript. IY and TT helped with laboratory investigations and revised the manuscript. TI, MTe, MTa, YT, and NA helped design the study and aided in sample collection. YO, SE, HK, and SM helped design the methodology of the study and were engaged in the production of antigens. KM helped with the laboratory investigations and edited the manuscript. All authors have reviewed and approved the final manuscript.
