# Corrigendum: Antigen-specific cytokine profiles for pulmonary *Mycobacterium avium* complex disease stage diagnosis

**DOI:** 10.3389/fimmu.2023.1275349

**Published:** 2023-08-24

**Authors:** Yoshiro Yamashita, Ikkoh Yasuda, Takeshi Tanaka, Toru Ikeda, Mayumi Terada, Masahiro Takaki, Yoshiko Tsuchihashi, Norichika Asoh, Yukiko Ohara, Shymaa Enany, Haruka Kobayashi, Sohkichi Matsumoto, Konosuke Morimoto

**Affiliations:** ^1^ Department of Clinical Medicine, Institute of Tropical Medicine, Nagasaki University, Nagasaki, Nagasaki, Japan; ^2^ Department of Respiratory Medicine, Shunkaikai Inoue Hospital, Nagasaki, Nagasaki, Japan; ^3^ Department of General Internal Medicine and Clinical Infectious Diseases, Fukushima Medical University, Fukushima, Fukushima, Japan; ^4^ Infection Control and Education Center, Nagasaki University Hospital, Nagasaki, Nagasaki, Japan; ^5^ Department of Respiratory Medicine, Nagasaki Rosai Hospital, Sasebo, Nagasaki, Japan; ^6^ Department of Internal Medicine, Koseikai Nijigaoka Hospital, Nagasaki, Nagasaki, Japan; ^7^ Department of Respiratory Medicine, Juzenkai Hospital, Nagasaki, Nagasaki, Japan; ^8^ Department of Bacteriology, Niigata University Graduate School of Medicine, Niigata, Niigata, Japan; ^9^ Department of Microbiology and Immunology, Faculty of Pharmacy, Suez Canal University, Ismailia, Egypt; ^10^ Biomedical Research Department, Armed Force College of Medicine, Cairo, Egypt; ^11^ Department of Respiratory Infectious Disease, Institute of Tropical Medicine, Nagasaki University, Nagasaki, Nagasaki, Japan

**Keywords:** *Mycobacterium avium* complex disease, clinical stage, *Mycobacterium avium*-associated antigens, cell-mediated immunity, CD4+T cells, CD19+B cells, cytokine profile

In the published article, there was an error in affiliation for author Shymaa Enany. Instead of “Department of Bacteriology, Niigata University Graduate School of Medicine, Niigata, Niigata, Japan”, it should be “Department of Microbiology and Immunology, Faculty of Pharmacy, Suez Canal University, Ismailia, Egypt & Biomedical Research Department, Armed Force College of Medicine, Cairo, Egypt”.

The authors apologize for this error and state that this does not change the scientific conclusions of the article in any way. The original article has been updated.

