# Supplementary material for: Antigen-specific cytokine profiles for pulmonary Mycobacterium avium complex disease stage diagnosis
Source: Front Immunol. 2023 Jul 14;14:1222428. doi: 10.3389/fimmu.2023.1222428 (PMC10380938; doi:10.3389/fimmu.2023.1222428)
Supplement: Supplementary file 1 [file DataSheet_1.docx]

Supplementary Material

**Antigen-specific cytokine profiles for pulmonary *Mycobacterium avium* complex disease stage diagnosis**

Yoshiro Yamashita, Ikkoh Yasuda, Takeshi Tanaka, Toru Ikeda, Mayumi Terada, Masahiro Takaki, Yoshiko Tsuchihashi, Norichika Asoh, Yukiko Ohara, Shymaa Enany, Haruka Kobayashi, Sohkichi Matsumoto, Konosuke Morimoto

*** Correspondence:** Yoshiro Yamashita E-mail: yoshiro@dg7.so-net.ne.jp

# Supplementary Data

**1.1 Reagents**

The following primers were used to amplify MAV0986, MAV1160, MAV1276, and MAV4925 by polymerase chain reaction (PCR) to allow the expression of C‐terminal, 6 × histidine-tagged recombinant proteins following ligation into the Nde1 and HindIII restriction enzyme sites of pET‐22b(+) (Novagen, WI, USA).

MAV_0986-Fw: GGGCATATGAAAACCGTTGCGGTGCGGCCGG

MAV_0986-Rv: CCCAAGCTTGCTCAGCGCGGGAATGATCTCCCGC

MAV_1160-Fw: GGGCATATGCTGGACATCCTGCCGTCGCTGG

MAV_1160-Rv: CCCAAGCTTCCCGCGGTCGCGGGCCAGCAGG

MAV_1276-Fw: GGGCATATGGCTGAAGCCGTCATCGTCGAG

MAV_1276-Rv: CCCAAGCTTCAACAGTTCCACGATGGTGG

MAV_4925-Fw: GGGCATATGCGGTGCGGCCCGCTGAACAC

MAV_4925-Rv: CCCAAGCTTCTTGTACAGGTCCGTGTGGTC

The resulting plasmids were introduced into ClearColi BL21(DE3) (Lucigen, WI, USA) by electroporation according to the manufacturer’s instructions, and transformants were selected by culturing on LB agar containing 50 µg/ml carbenicillin at 37 °C. Each recombinant ClearColi containing the expression plasmid was grown in 500 ml of LB medium in a Sakaguchi flask at 37 °C with horizontal shaking at 120 rpm and cultured until reaching an optimal density of 0.5 at 600 nm. For induction of recombinant *Mycobacterium avium* proteins, isopropyl-1- thio-beta-D-galactopyranoside was then added at a final concentration of 0.5 mM and further cultured for 1 h at the same setting. Bacteria were then immediately cooled on ice and collected by centrifugation at 7,000 × *g* for 10 min. Bacteria were then lysed by rotating in 10 ml of BugBuster Protein Extraction Reagent (Sigma, St. Louis, MO, USA) containing benzonase and lysozyme for 15 min at RT. Lysed bacterial samples were then centrifuged at 10,000 × *g* for 15 min, and the solubilized proteins were obtained as supernatants. Recombinant proteins in the supernatants were purified using an Ni-NTA column, as previously described [1]. The purity of the proteins was confirmed by SDS-PAGE analysis and stained with Coomassie Brilliant Blue R-250 (CBB) as a single band, as shown in Supplementary Figure 1.

The following fluorescently labeled monoclonal antibodies were used: anti-CD3-APC-Cy7 (HIT3a), anti-IFN-γ-PE-Cy7 (4S. B3), anti-IL-10-PE (JES3-9D7), anti-IL-17-Alexa Fluor 700 (BL168), anti-TNF-α-PerCP-Cy5.5 (MAb11) (Biolegend, San Diego, CA, USA), anti-CD4-Pacific Blue (OKT4), anti-IL-2-APC (MQ1-17H12), anti-IL-13- FITC (PVM13-1) (eBioscience, San Diego, CA, USA), and anti-CD19-ECD (J3-119) (Beckman Coulter, Brea, CA, USA). Cell viability was assessed using a LIVE/DEAD kit (Invitrogen, Carlsbad, CA, USA). The FcR blocking reagent was purchased from MBL (Nagoya, Japan). The CD28/CD49d co-stimulator was purchased from BD Biosciences (San Jose, CA, USA). Brefeldin-A and Monensin sodium salt were purchased from Sigma-Aldrich (St. Louis, MO, USA) and Wako Junyaku Co., Ltd.(Tokyo, Japan), respectively.

**1.2 References**

1. Aoki K, Matsumoto S, Hirayama Y, Wada T, Ozeki Y, Niki M, *et al*. Extracellular mycobacterial DNA-binding protein 1 participates in mycobacterium-lung epithelial cell interaction through hyaluronic acid. J Biol Chem (2004) 279:39798-39806.

**1.3 Figure legends**

**Supplementary Figure 1.**

Protein antigens used to stimulate peripheral blood mononuclear cells (PBMCs). Purified MAV0986 (lane 1), MAV1160 (lane 2), MAV1276 (lane 3), and MAV4925 (lane 4) were fractionated by SDS-PAGE and stained with CBB. M, protein size markers. One microgram of each recombinant protein was applied to 15 % polyacrylamide gel.

**Supplementary Figure 2.**

(A) Gating strategy for the flow cytometric analysis of CD4+T cells and CD19+B cells. Peripheral blood mononuclear cells (PBMCs) were isolated from blood samples. The cell surface was stained with fluorescently labeled monoclonal antibodies against CD3 (APC-Cy7), CD4 (Pacific Blue), and CD19 (ECD). Intracellular staining was performed with fluorescently labeled monoclonal antibodies against IFN-γ (PE-Cy7), IL-2 (APC), TNF-α (PerCP-Cy5.5), IL-17 (Alexa Fluor 700), IL-10 (PE), and IL-13 (FITC). Data were collected with Gallios and analyzed using FlowJo software. Lymphocytes were identified by scatter properties (forward scatter × side scatter plot) and dead cells were excluded. The surface CD3+ cells represent T lymphocytes and the surface CD19+ cells represent B lymphocytes. CD4 + cells were chosen from the T lymphocytes. (B) Representative flow cytometry results showing IFN-γ, IL-2, TNF-α, IL-17, IL-10, and IL-13 responses of CD3+CD4+ cells to Staphylococcus endotoxin b stimulation. (C) Representative flow cytometry results showing TNF-α responses of CD3+CD4+ cells to various pulmonary MA*-*associated antigens.
