# Supplementary figures and images for: Antigen-specific cytokine profiles for pulmonary Mycobacterium avium complex disease stage diagnosis
Source: Front Immunol. 2023 Jul 14;14:1222428. doi: 10.3389/fimmu.2023.1222428 (PMC10380938; doi:10.3389/fimmu.2023.1222428)

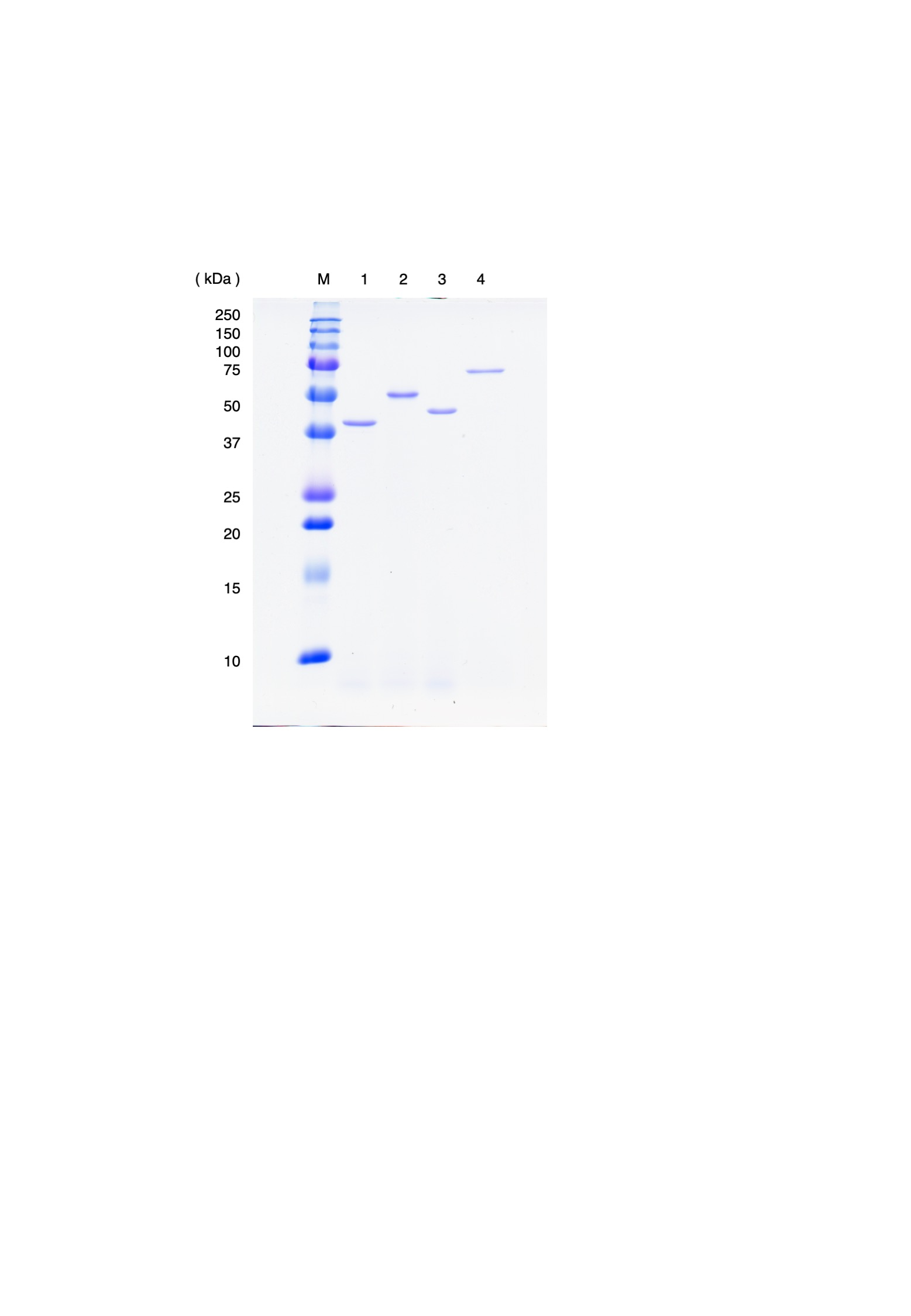

Supplement: Supplementary file 2 [file Image_1.jpg]

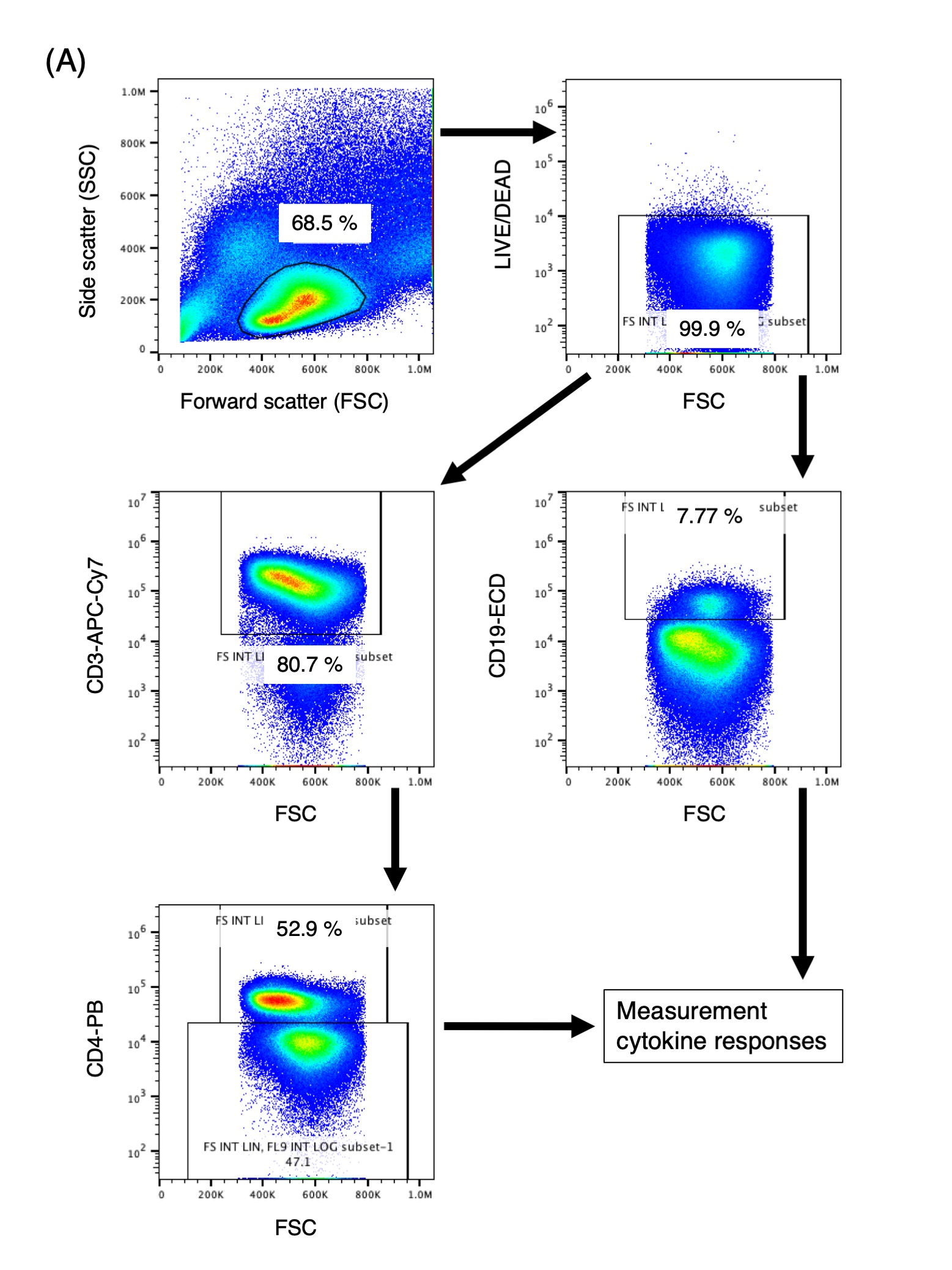

Supplement: Supplementary file 3 [file Image_2.jpeg]

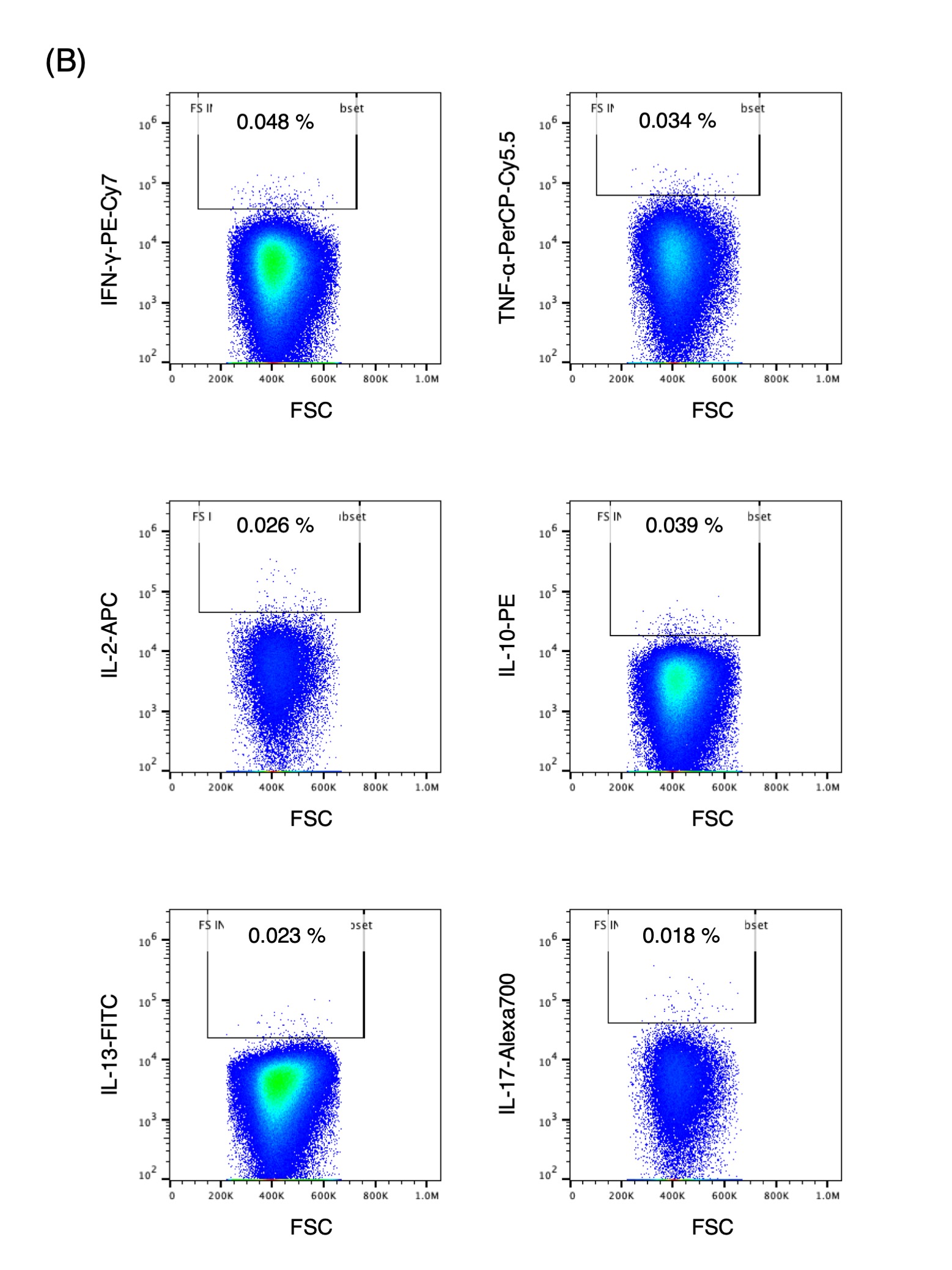

Supplement: Supplementary file 4 [file Image_3.jpeg]

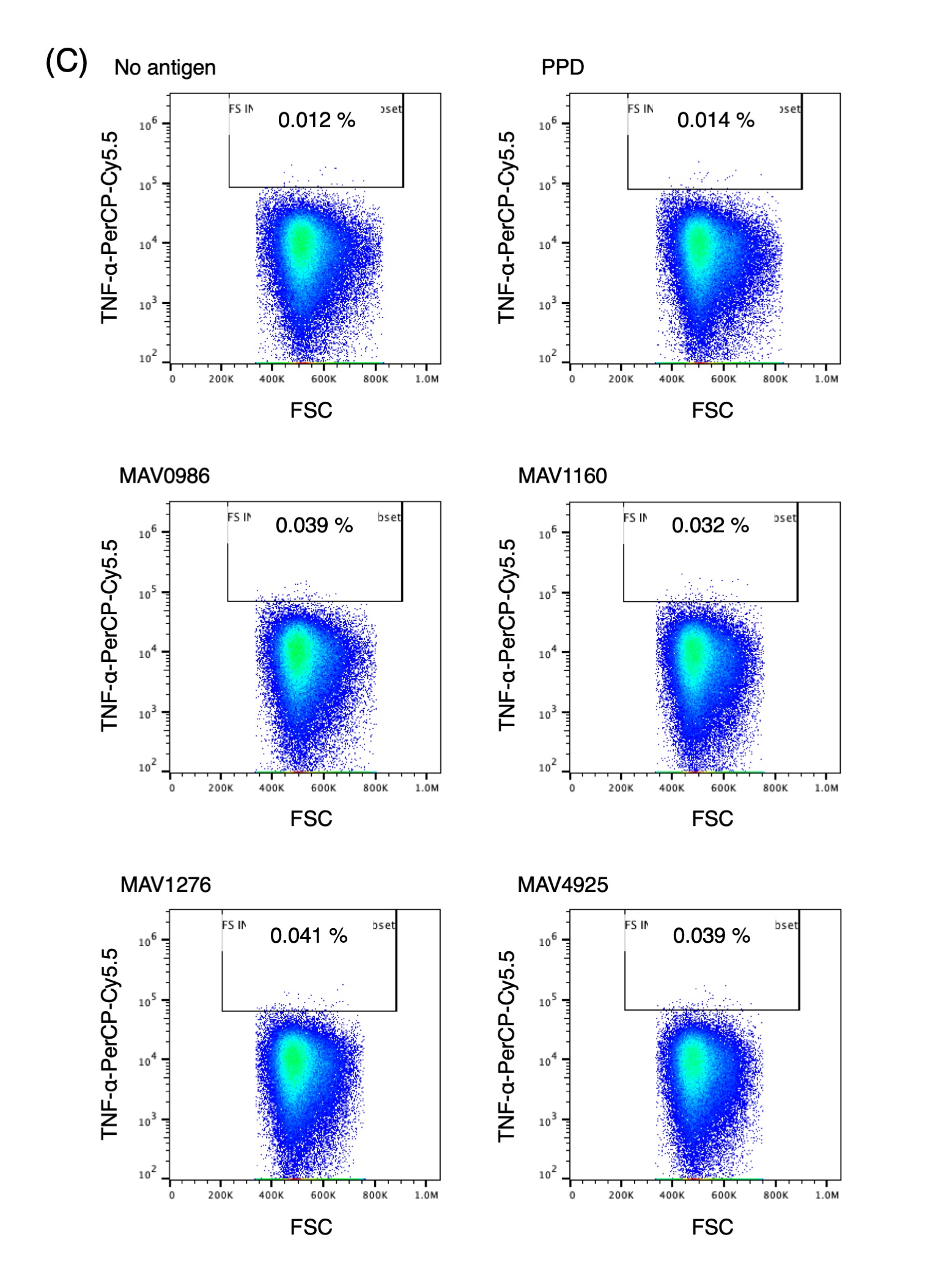

Supplement: Supplementary file 5 [file Image_4.jpeg]
